# Electromigration in Gold Films on Flexible Polyimide Substrates as a Self-healing Mechanism

**DOI:** 10.1080/21663831.2015.1105876

**Published:** 2015-10-27

**Authors:** Barbara Putz, Oleksandr Glushko, Megan J. Cordill

**Affiliations:** ^a^ Erich Schmid Institute of Materials Science, Austrian Academy of Sciences and Department of Materials Physics, Montanuniversität Leoben, Jahnstrasse 12, Leoben8700, Austria

**Keywords:** Thin Films, Electromigration, Self-healing, Flexible Electronics

## Abstract

The study of electromigration (EM) in metallisations for flexible thin film systems has not been a major concern due to low applied current densities in today's flexible electronic devices. However, the trend towards smaller and more powerful devices demands increasing current densities for future applications, making EM a reliability matter. This work investigates EM in 50 nm Au thin films with a 10 nm Cr adhesion layer on a flexible polyimide substrate at high current densities. Results indicate that EM does occur and could be used as a self-healing mechanism for flexible electronics.

## GRAPHICAL ABSTRACT

**Figure:**
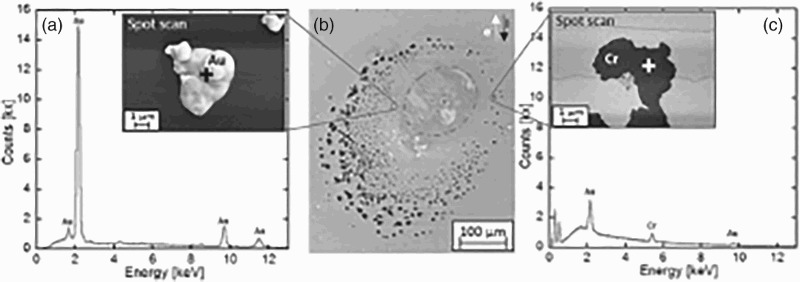

## Introduction

Flexible electronics are a new and promising technology with applications as diverse as rollable displays or artificial skin.[1–3] The devices utilise thin metal films for electrical connections on compliant polymer substrates. In today's flexible electronic devices, the current densities are in the range of 5 × 10^4^ A/cm^2^. However, the trend for smaller and more powerful devices will move towards higher current densities. Thereby electromigration (EM), a current-driven self-diffusion of metal atoms, can become an issue. At sufficiently high current densities and operational times, structural or surface changes in current-carrying components due to migrating metal atoms can cause the loss of current-carrying capability or the occurrence of electrical short circuits.[4] EM can typically occur in metal films at current densities of 10^4^–10^6^ A/cm^2^.[5–7] Moving electrons force nominally stationary metal atoms to move in the same direction as the electron flow. Accordingly, the structure of the conductor changes with an accumulation of material (hillocks) at the positive pole and depletion (voids) towards the negative pole.[4,8] Detailed information about the physical principles of EM can be found in [4,9,10]. EM is diffusion controlled and strongly fostered by elevated temperatures and fine microstructures. Thin films are especially prone to EM, as their microstructure is composed of a large number of rapid low-temperature diffusion paths, such as grain boundaries and interfaces that allow significant mass transport.[11] Diffusion processes along surfaces and interfaces have been shown to become more pronounced with decreasing film thickness.[11] Therefore, the threshold current density when EM occurs can be considerably lower than 10^6^ A/cm^2^.

A tremendous amount of effort has focused on how to impede EM in conventional rigid microelectronic devices.[4,8,11,12] Conventional EM testing utilises the drift test, a set-up introduced by Blech in 1976.[9,13] In practice, accelerated EM tests based on Black's Law [8,14] are performed. Data obtained at high current densities and elevated temperatures are extrapolated to long-term in-use conditions, where current densities and temperatures are typically lower, to assess the long-term reliability of microelectronic devices. The literature also reports about alternating current experiments and reversible EM phenomena.[13,15,16]

In contrast, the study of EM in flexible film systems is a relatively new field. Besides EM, several other effects need to be taken into account while operating with high current densities on polymer substrates. Low thermal stability and local melting of the polymer substrate due to temperature increase and Joule heating [17] exacerbate electrical testing on flexible substrates. This current work investigated EM in thin Au films on a flexible polyimide (PI) substrate; both materials are commonly used in flexible electronics. A custom-made experimental set-up was utilised and introduced since the Blech structure,[9] required for the conventional drift test on rigid substrates, was unfeasible for the film system under investigation. The end goal was to determine if EM occurs in flexible electronics which were subjected to mechanical damage similar to what would be induced during service.

## Experimental procedure

The 50 nm Au films were sputter deposited onto a 50 µm-thick flexible PI substrate (Kapton) in a similar manner to Yeager *et al*. [18]. A 10 nm Cr interlayer was deposited prior to Au deposition without breaking vacuum. Sputtering was performed with a DC Magnetron system. The deposition parameter for the Cr layer was an Ar gas pressure of 1.5 × 10^−3^ Torr at 100 W DC power. Sputtering of the Au film was performed with Ar at a pressure of 7.5 × 10^−3^ Torr at a power of 75 W DC. The Au films had a nanocrystalline microstructure with a grain size between 20 and 50 nm measured using transmission electron microscopy.[19]

**Figure 1.**
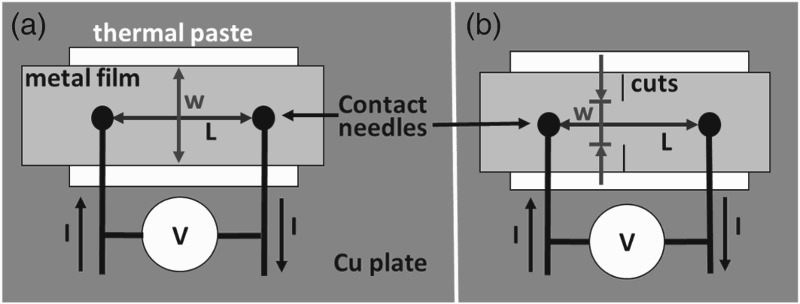
Experimental set-up for EM testing. (a) Geometry 1 and (b) Geometry 2 utilises cuts to lower the applied current while keeping the same current density as for (a).

Rectangular samples sized 7 mm × 30 mm were uniaxially strained with an Anton Paar TS600 straining stage to a maximum strain of 15% to generate a repeatable crack pattern in the Au film with through thickness cracks (TTCs) perpendicular to the straining direction. Detailed information about the mechanical behaviour of the Au films and the influence of the Cr layer are published elsewhere.[20] After straining the samples were cut into thin strips (3 mm × 30 mm, Geometry 1, Figure 1(a)) and subjected to high current densities with the TTCs oriented perpendicular to current direction to determine the effects of the EM on films containing a regular array of TTCs. A second sample geometry was introduced to locally increase the current density and to better examine the self-healing of cracks. Cuts were made on both sides of a sample, leaving a width of 0.5 mm to 1 mm between the cuts (Geometry 2, Figure 1(b)). The cuts narrowed the area of interest for the observation of crack closure and allowed higher current densities and less Joule heating.

EM testing was performed utilising a standard test configuration. Two needles contact the sample surface applying constant direct current through the film using a Keithley 2400 SourceMeter. The film resistance was measured using Ohm's law and recorded over time. The distance between the contact needles was approximately *L* = 3 mm for all experiments. The complete set-up was placed on a copper plate to allow fast heat dissipation. A thermal paste (Electrolube, HTCP) was applied under the tested region of the sample to increase the thermal stability and prevent melting of the polymer substrate. The current density, *J*, was calculated using 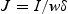 with the nominal gold film thickness *δ* = 50 nm and *I* is the applied current. The 10 nm Cr layer was neglected due to its relatively high resistivity. For Geometry 1 the width of the sample, *w*, was taken to be the mean width of the sample between the contacts. For Geometry 2, *w* equals the length between the two cuts.

At the beginning of an experiment, the current was increased stepwise to a pre-defined testing current density that was then held constant during the experiments. The testing current density was chosen in such a way that no immediate decomposition (melting) of the polymer substrate occurred (upper limit) but to obtain potential EM effects at reasonable times (lower limit). A compromise between those two criteria had to be found for each tested sample. The upper limits for immediate substrate melting were experimentally determined and were sensitive to slight changes in the testing conditions. Seven samples were subjected to current densities ranging from 0.56 to 0.86 MA/cm^2^ for times between 1 h and 24 h. Sample temperatures during testing were estimated using the temperature dependence of the electrical resistance [21] and were less than 100°C.

After electrical testing and prior to examination with scanning electron microscopy (SEM), the samples underwent an ultrasonic cleaning treatment to remove the thermal paste. To investigate surface changes obtained during testing, atomic force microscopy (AFM, Veeco Dimension 3100) and energy dispersive spectroscopy (EDS) analysis (SEM LEO 1525) were performed. Additionally, SEM images of strained films were taken before and after electrical testing of the same area (Geometry 2) for comparison and crack spacing measurements.

## Results and discussion

**Figure 2.**
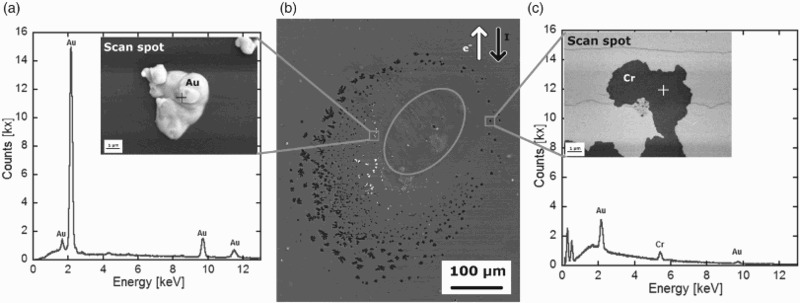
Surface modifications obtained at *J* = 0.7 MA/cm^2^ after 6.3 h. The position of the contact needle is marked with a dashed circle in the overview micrograph in (b). A ring of voids around the contact with hillocks inside this ring can be seen. Technical and physical current directions are indicated with arrows. Representative EDS scans of surface modifications and SEM micrographs of (a) Au hillock and (c) void.

An average current density of 0.7 MA/cm^2^ was applied to the strained Geometry 1 samples for times between 1 and 8 hrs while recording the resistance. No significant change in the resistance was recorded at the tested current density and time ranges. For Geometry 1 surface modifications occurred only close to the contact needles (Figure 2). Considering the sample geometry and current flow lines, the current density is the highest close to the contacts. Therefore, the position of the surface modifications is reasonable in terms of EM considerations. In the literature this stage is referred to as early stage of EM.[22] With the aid of scanning tunnelling microscopy Panin et al. [23] found that in 100 nm-thick Au thin films on rigid glass ceramic substrates the first surface topography changes begin to manifest themselves after a few minutes at comparable current density levels.

An example of surface modifications near the contact is shown in Figure 2. The sample depicted was subjected to an average current density of *J* = 0.7 MA/cm^2^ for 6.3 h. Figure 2(b) is an overview micrograph of the whole contact area, where one needle contacted the sample surface and is indicated with a dashed circle. Black and white arrows indicate the technical and physical current directions, respectively. What can be observed is that a ring of voids (holes) has formed around the contact. The width of the ring increases in the direction of the second contact. Inside the ring of voids, Au hillocks can be found. In Figure 2(a) and 2(c), magnified images of an Au hillock and a void can be seen, respectively. The visible cracks in both micrographs stem from the initial straining. According to the phase diagram Au–Cr, [24] no phase transition occurs in the present temperature regime of 25–95°C that could explain the modifications as intermetallic phases.

EDS spot scans revealed the chemical composition of the voids and hillocks. Representative scans can be seen in Figure 2(a) and 2(c), where the scan position is marked with a cross. Hillocks inside the ring of voids consist of pure Au (spot scan Figure 2(a)), whereas particles outside are contaminations from the thermal paste or atmosphere. Spectra of voids show Au and Cr peaks (Figure 2(c)). Au, as a heavy element, absorbs the low-energetic X-ray radiation coming from light elements of the PI substrate underneath. The 10 nm Cr layer was neglected in absorption considerations due to its low thickness. The Au peaks in the void spectra result from the fact that the lateral resolution of EDS is in the range of a few µm (depending on the size of the interaction volume generated by the primary electron beam).

**Figure 3.**
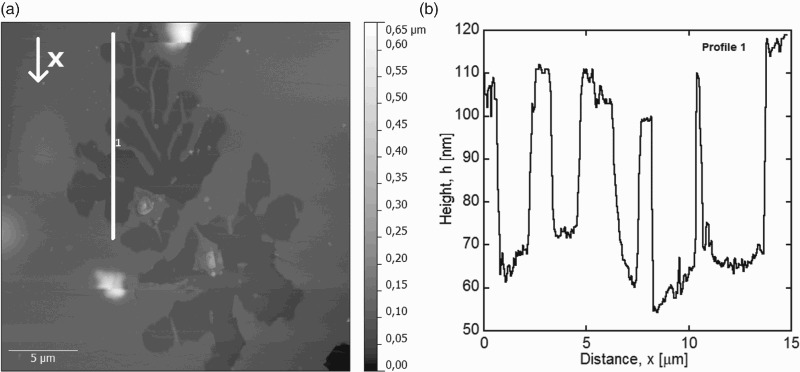
AFM height image of a void (a) and corresponding extracted height profile (b). The position of the extracted profile (white line) and *x*-direction (arrow) are indicated in (a). The mean depth of the voids is 40–50 nm.

AFM investigations confirmed that voids were indeed missing the gold film. Figure 3 shows an AFM height image of a hole and an extracted height profile. The position of the profile is indicated by the white line in Figure 3(a). The profile reveals a mean depth of the holes of 40–50 nm. This implies that part or the entire 50 nm gold film is missing and formed the hillocks.

**Figure 4.**
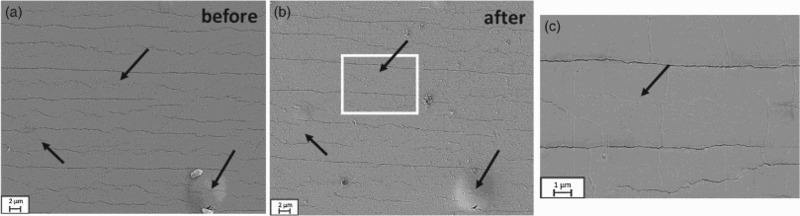
SEM micrographs of the same area of a cracked sample (a) before and (b) after subjecting the film to *J* = 0.6 MA/cm^2^ for 24 h. (c) Higher magnification of box in (b) further illustrating that cracks have closed. Arrows indicate where fine cracks are no longer visible.

Experiments performed on Geometry 1 clearly demonstrated that EM can occur on flexible substrates. In order to determine if the EM observed at the contacts could be utilised to close cracks, Geometry 2 was tested. This sample geometry allowed for a more local application of the current, less Joule heating, and the possibility to examine the same area before and after electrical exposure. Figure 4 shows SEM micrographs of a cracked film taken from the same area before and after electrical testing of a sample with the cut geometry. The sample presented here was subjected to a current density of *J*  =  0.6 MA/cm^2^ for 24 h. The number of visible cracks decreased after the application of the high current density (crack spacing increased). Black arrows mark areas where fine cracks are no longer visible (Figure 4(c)). Long through thickness cracks are still clearly visible after electrical testing. This result suggests that there is a certain maximum crack depth and width at which closure of the cracks through electromigrative mass diffusion became impossible under the applied conditions. To quantify the amount of crack closure, the crack spacing *λ* (distance between two cracks) was determined for each micrograph before and after electrical testing. The average crack spacing in Figure 4(a) and 4(b) micrographs increased from (2.4 ± 0.6) µm before to (3.6 ± 1) µm after electrical testing. Voids and hillocks were not observed near the cracks nor at the needle contacts because the applied currents were lower, but voids were found between the cuts and cracks in other samples (see Figure S1).

## Conclusions

Surface topography modifications to Au on PI during electrical testing indicate that EM, namely the evolution of Au hillocks and voids, is possible in initially cracked 50 nm Au films with a 10 nm Cr interlayer on flexible PI at current densities on the order of 10^6^ A/cm^2^. The large surface modifications occurred close to the contact needles, a reasonable position with the highest local current density considering current flow lines and sample geometry. EDS and AFM analyses revealed the chemical composition and topography of the surface changes, respectively. More subtle surface modifications found away from the contact needles in the form of an increase in crack spacing after electrical testing point towards a self-healing mechanism in flexible electronics. The obtained results demonstrate that EM does occur in flexible electronic film systems and indicates the possibility of harnessing this process as a self-healing mechanism with further research.

Supplementary Material.docx
