# Supplementary material for: Electromigration in Gold Films on Flexible Polyimide Substrates as a Self-healing Mechanism
Source: Mater Res Lett. 2015 Oct 27;4(1):43–7. doi: 10.1080/21663831.2015.1105876 (PMC4854219; doi:10.1080/21663831.2015.1105876)
Supplement: Supplementary Material.docx [file tmrl_a_1105876_sm3152.docx]

# Electromigration in gold films on flexible polyimide substrates as a self-healing mechanism

Barbara Putz, Oleksandr Glushko, and Megan J. Cordill^*^

Erich Schmid Institute of Materials Science, Austrian Academy of Sciences and Department of Materials Physics, Montanuniversität Leoben, Jahnstrasse 12, Leoben 8700, Austria

Tele: +43 3842 804 112

*corresponding author: megan.cordill@oeaw.ac.at

**Supplementary Figure**

**
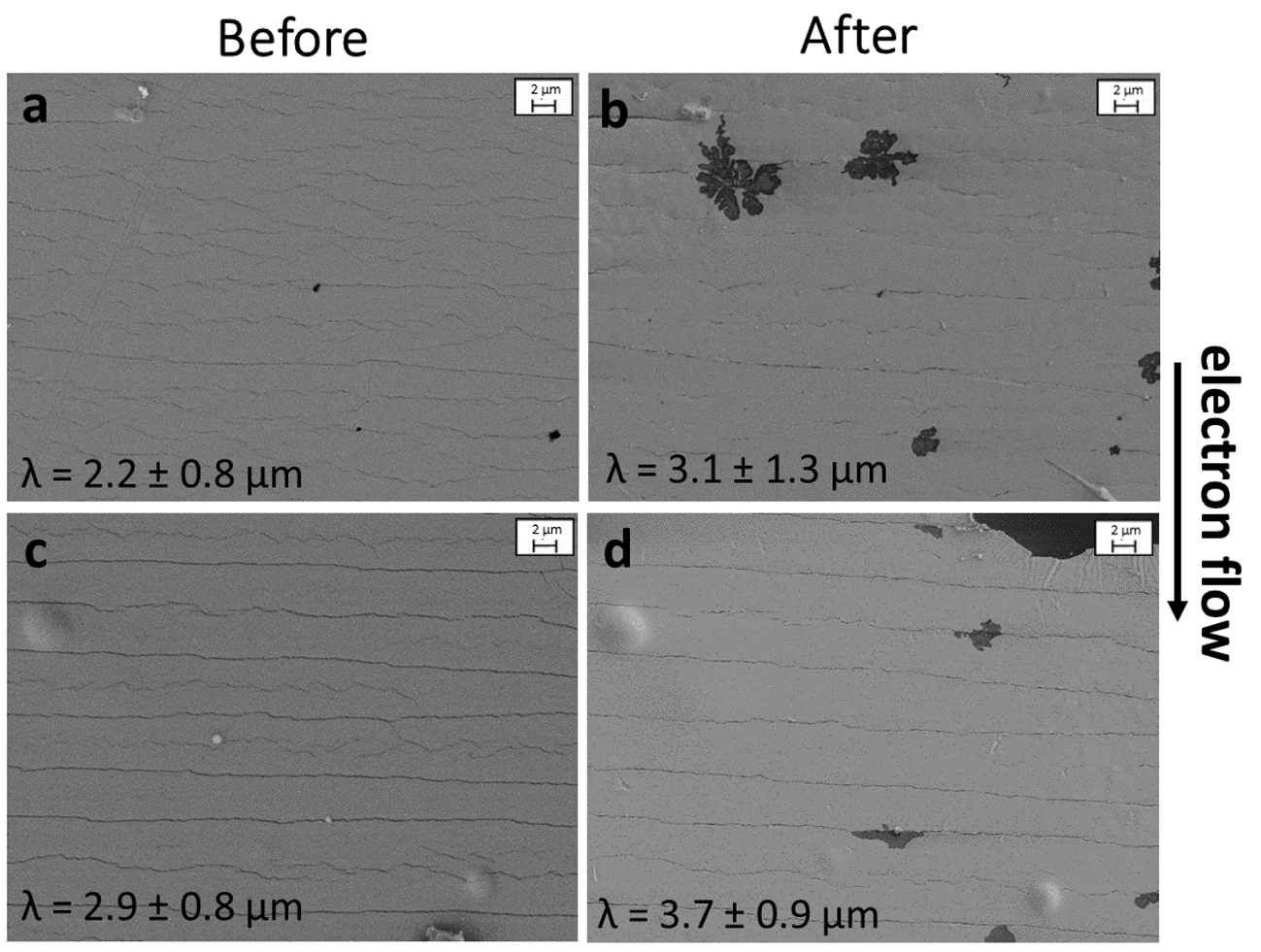
**

Figure S1: Further examples of crack closure (self-healing) induced by electromigration. (a) and (c) before electrical testing and (b) and (d) after application of 0.7 MA/cm^2^ current density for 4 hours. The average crack spacing of each image shown in bottom left corner and increase after the electrical testing indicating crack closure. Voids are also visible in both after micrographs.
